# Longitudinal changes of health-related quality of life over 10 years in breast cancer patients treated with radiotherapy following breast-conserving surgery

**DOI:** 10.1007/s11136-023-03408-y

**Published:** 2023-04-24

**Authors:** Yifeng Gao, Juan C. Rosas, Hanna Fink, Sabine Behrens, Jenny Chang-Claude, Petra Seibold

**Affiliations:** 1 Division of Cancer Epidemiology, German Cancer Research Center (DKFZ), Im Neuenheimer Feld 581, 69221, Heidelberg, Germany; 2 Institute for Medical Information Processing, Biometry and Epidemiology, Ludwig-Maximilians-University, Munich, Germany; 3 University Cancer Center Hamburg, University Medical Center Hamburg-Eppendorf, Hamburg, Germany

**Keywords:** Breast cancer, Survivorship, Health-related quality of life, Adjuvant radiotherapy, Long-term symptoms, Sleep problems

## Abstract

**Purposes:**

The study intended to (1) assess changes of health-related quality of life (HRQoL) between early treatment-related time points and 10 years post-treatment in a cohort of breast cancer (BC) patients who received radiotherapy (RT), (2) to evaluate differences in HRQoL between long-term BC survivors and unaffected women from the same geographical region and (3) to identify determinants of long-term HRQoL in the survivor cohort.

**Methods:**

292 BC patients were recruited prior to RT after breast-conserving surgery between 1998 and 2001 in Germany and prospectively followed up for a median of 11.4 years (range 10.3–12.8 years). HRQoL was assessed using EORTC QLQ-C30 at pre-RT (baseline), during RT, 6 weeks after RT, and at the 10-year follow-up. Changes in mean HRQoL scores over time were assessed using linear-mixed models. HRQoL in long-term survivors and controls was compared using Wilcoxon rank-sum test, stratified by age groups. Multivariable linear regression models were used to identify determinants for HRQoL in long-term BC survivors.

**Results:**

Compared to baseline level (mean summary score of 64.9), global health status/quality of life (GHS/QoL) declined during RT (62.4) and improved 6 weeks after RT (69.9) before decreasing to baseline level at the 10-year follow-up (66.7). Most functional domains deteriorated or remained stable at 10 years post-diagnosis compared to post-RT scores, except for role functioning which improved, while dyspnea and diarrhea significantly deteriorated between those two time points. There were no significant differences in long-term GHS/QoL between BC survivors 10 years post-RT and controls for all age groups (*p* > 0.05). However, deficits in specific HRQoL domains such as emotional burden, sleep problems or fatigue were found to more strongly affect survivors, in particular those younger than 65 years, compared to controls. In the determinant analysis, being overweight was associated with lower GHS/QoL and physical functioning, while living with others was found to be associated with better physical functioning, and decreased dyspnea and pain levels. Certain comorbidities such as depression had a strong association with multiple HRQoL domains, including lower GHS/QoL and functioning as well as a higher level of fatigue, pain, sleep/intestinal problems, and financial difficulties. Side effects such as lymphedema/pain and fibrosis were associated with worse physical and social functioning, respectively.

**Conclusion:**

The long-term GHS/QoL remained comparable when compared with the control population while restrictions in certain functional and symptoms domains in long-term BC survivors persisted over 10 years, in particular among younger survivors. Targeted screening to identify cancer survivors at risk for psychosocial/other impairment accounting also for comorbidities and treatment side effects may be warranted in long-term aftercare to address unmet health needs.

**Supplementary Information:**

The online version contains supplementary material available at 10.1007/s11136-023-03408-y.

## Plain English summary

Late effects after cancer treatment might lead to impaired health-related quality of life in cancer survivors. However, knowledge on quality of life among long-term breast cancer survivors is limited. This study describes the time changes of health-related quality of life during the treatment period as well as after 10 years in a unique cohort of breast cancer survivors from Germany who were treated with radiotherapy after surgery without chemotherapy. This study found that the self-reported quality of life decreased during radiotherapy but improved after cancer treatment. Ten years after cancer diagnosis, global quality of life was comparable to that of an unaffected control population, yet deficits in specific fields such as sleeping problems, fatigue, and emotional burden persisted for more than a decade, in particular in younger survivors and patients with certain comorbidities and/or treatment-related side effects. Findings from this study suggest more comprehensive long-term follow-up care may be warranted, with specific focus to identify psychosocial and other unmet health needs.

## Introduction

Prevalence of breast cancer (BC) survivors is increasing with 5-year and 10-year survival rates of 79% and 66%, respectively, due to earlier detection and improved cancer treatments [1–3]. Although BC prognosis has improved in recent years, late effects, such as psychosocial problems, insomnia, or other symptoms may still persist years after treatment [4, 5]. BC and treatment-related symptoms are important dimensions of health-related quality of life (HRQoL) and can adversely affect HRQoL of cancer survivors. Thus, the investigation of HRQoL of long-term BC survivors has become a major interest of BC survivorship studies.

Previous studies have shown that many BC survivors may feel unable to cope with life, family, and work expectations due to reduced physical, emotional, or cognitive capacity years after treatment [6–9]. Although overall HRQoL in medium/long-term (≤ 10 years) and also very-long-term survivors (> 20 years) has been reported to be comparable to a control population, restrictions in certain functional performances, and symptoms persisted [4, 10–13]. Fatigue, sleeping problems, cognitive problems, physical dysfunctions, and pain were shown to be long-lasting symptoms in medium-term (< 5 years) survivors [4, 14] and were similarly found in a cross-sectional study of very long-term BC survivors 14–24 years after diagnosis [13]. Restrictions of HRQoL were consistently found to be more common among younger survivors [15–18] who usually reported a higher level of emotional burden and were more worried about financial problems [18, 19]. Some studies showed that both tumor and treatment-related factors such as adjuvant chemotherapy [20–24], hormone therapy, as well as lifestyle factors such as obesity [25] were associated with lower HRQoL even years after diagnosis. Depression was found to be highly associated with worse psychosocial problems and impaired HRQoL [26].

Follow-up care for BC survivors usually ends at around 5 years past diagnosis [27–29]. Studies of HRQoL in BC survivors with long-term follow-up more than 10 years are rare. Therefore, we took advantage of the data prospectively collected as a cohort of BC patients who were treated with radiotherapy (RT) after breast-conserving surgery and followed up for 10 years. We aimed to (1) examine changes in mean HRQoL scores between early treatment measurements and 10 years post-treatment, to (2) evaluate differences in HRQoL between long-term BC survivors and a control population of the same geographical region and to (3) identify determinants of long-term HRQoL in this specific cohort of breast cancer survivors.

## Methods

### Data collection and study population

#### Patient cohort

Female BC patients from a population-based prospective cohort study (ISE for “Individuelle StrahlenEmpfindlichkeit,” individual radiation sensitivity) in the Rhine-Neckar region of Germany with an original focus on individual radiation sensitivity were included in this analysis. The ISE study recruited 478 BC patients with a histologically confirmed diagnosis of in situ or primary invasive BC (all stages) at ages 26–87 years between 1998 and 2001. Patients received adjuvant whole breast RT in 25 fractions à 2 Gy ± boost at a total dose of 50–66 Gy after breast-conserving surgery [30]. Patients treated with chemotherapy prior or simultaneously to radiotherapy were not eligible for the study [31, 32]. Data on HRQoL were collected at baseline (t1), which was after breast-conserving surgery and before the beginning of post-operative RT, during RT (t2 at 36–42 Gy, t3 at 44–50 Gy), at the end of RT (t4), 6 weeks after RT (t5), and about 10 years after RT (mean: 11.4 years) (t10). At baseline, information on socio-demographics, lifestyle factors, comorbidities, and medical history was assessed by a self-administered questionnaire. The histological characteristics of the tumor including information on stage at diagnosis and treatment were obtained from pathology reports and hospital records, respectively. Patients were re-contacted in 2011 and visited for examination of late adverse effects of RT, disease progression as well as other related information. Information on recurrences and metastases were collected during the follow-up and were based on self-reports as well as medical records.

Of the initial 478 BC patients, 72 (19%) had passed away (due to BC or other causes) by 2011, 47 (10%) were excluded from further participation because of the original study design due to ablatio mammae, contralateral BC or chemotherapy they have undergone during the follow-up, 54 (11%) refused to participate or were not able to, 10 (2%) were not traceable, and 3 (0.6%) did not fill out the follow-up questionnaire on HRQoL, resulting in 292 (61%) BC survivors participating at the 10-year follow-up (Fig. S1).

All patients gave written informed consent. The ISE study was approved by the ethics committee of the University of Heidelberg.

#### Control population

Control persons were taken from a large population-based case–control study conducted between 2002 and 2005 to identify potential risk factors for the development of BC after menopause (MARIE study). Breast cancer patients aged 50–74 years at diagnosis and age-matched unaffected women (controls) were recruited from the two study regions, Rhine-Neckar-Karlsruhe and Hamburg [33, 34]. Controls were drawn from the population registries and were similarly followed up like the BC cases after 10 years in 2016 [33]. We included only the controls from the Rhine-Neckar-Karlsruhe study region in our analysis, as the ISE patient cohort was recruited from a very similar region. Information including socio-demographics, lifestyle behaviors and comorbidities was collected at recruitment in a personal interview. Controls were eligible for this analysis if EORTC C30 HRQoL data were available at the 10-year follow-up, amounting to 1680 individuals. Although controls were similarly followed-up compared to the patient cohort, quality of life data was not available at baseline.

The MARIE study was approved by the ethics committee of the University of Heidelberg, the Hamburg Medical Council, and the Medical Board of the State of Rhineland-Palatinate.

### Health-related quality of life measurement

HRQoL was prospectively assessed using the EORTC QLQ-C30 version 3.0 [35]. It is composed of 30 items: global health status/quality of life (GHS/QoL), five functional domains (physical, role, cognitive, emotional and social functioning), three combined symptom domains (pain, fatigue and nausea/vomiting) and six single symptom items (dyspnea, insomnia/sleep problems, appetite loss, constipation, diarrhea and financial difficulties). In accordance with the EORTC QLQ-C30 scoring manual, raw scores were calculated as arithmetic means if at least half of the items for the scale were answered, then linearly transformed into 0 to 100 scale [36]. Higher scores indicate a better functioning or a better GHS/QoL, whereas higher scores on the symptom scale represent a heavier burden of symptoms. Based on the original study design with focus on radiation toxicity, the HRQoL data was collected at baseline (pre-RT, after breast-conserving surgery), during RT, end of RT, 6 weeks after RT and at the 10-year follow-up.

### Statistical analysis

Linear-mixed models (LMM) were used to evaluate differences between selected time points while accounting for the correlation of multiple measurements within a subject (i.e., random intercept for subject). For this part of the analysis, all patients with HRQoL data at t10 were included as part of the regression models. The time points were included as a categorical variable to test mean HRQoL score differences between the time points t1, t5 and t10. All models were adjusted for age at baseline. In addition to LMM, linear regression models were used to evaluate the specific effect of baseline age on mean score differences between 6 weeks after RT (t5) and 10 years after (t10) for those HRQoL domains with significant differences at t10 vs. t5. Wilcoxon rank-sum test was used to assess differences between patients and controls stratified by age groups (< 65, 65– < 75, ≥ 75 years) at the 10-year follow-up. For the last objective, multivariable linear regression models were obtained to identify determinants for HRQoL domains at the 10-year follow-up in BC survivors. Potentially relevant factors including age (< 65, 65– < 75, > 75), BMI (≥ 25 vs. < 25 kg/m^2^), living with others, smoking status (never, former, current smokers), endocrine therapy (tamoxifen, aromatase inhibitors), recurrence status (yes/no) and selected comorbidities (depression, hypertension, stroke and diabetes) and symptoms (pain/lymphedema and fibrosis) assessed at follow-up were analyzed in these models.

All statistical analyses were performed using R Version 4.1.0 (R core team 2021, Vienna, Austria). A *p* value < 0.05 (two-sided) was considered statistically significant.

## Results

Out of 478 women with BC who were initially recruited prior to post-operative RT, 292 patients participated in the 10-year follow-up (Fig. S1). Study characteristics (baseline, follow-up) are shown in Table 1. Compared to all 478 patients, the 292 long-term survivors participating in the 10-year follow-up had initially reported less comorbidities, higher education level, and a more favorable tumor stage at baseline. The mean age of the participants was around 2 years less than that of the patients not participating in the follow-up.

**Table 1:** Study characteristics of the breast cancer patient cohort at baseline and 10-year post-diagnosis and the control population at the 10-year follow-up

	Patient Cohort (Southwest Germany)													Control Population (Southwest Germany)*		
	Patients who attended baseline						Patients who attended 10-year follow-up									
	At Baseline			Drop-Outs			At Baseline				10-year past diagnosis			10-year follow-up		
	*N* = 478		(%)	*N* = 186		(%)	*N* = 292			(%)	*N* = 292		(%)	*N* = 1680		(%)
Age (years)																
<50	49		10.3	12		6.5	37			12.7	1		0.3	0		0
50– < 65	286		59.8	82		44.1	204			70	68		23.3	100		4.6
65– < 75	102		21.3	61		32.8	40			13.7	145		49.7	811		46.4
75 +	31		6.6	24		12.9	7			2.4	78		26.7	763		48.6
mean (SD)	61.0		9.0	64.3		10.0	59.0			7.6	70.4		7.7	73.8		5.6
range	26.70–87.39			26.70–87.39			36.74–80.04				48.35–91.41			61.92–88.07		
BMI (kg/m^2^)																
< 25	236		49.4	84		45.2	152			52.1	136		46.6	732		43.6
> = 25	231		48.3	94		50.5	137			46.9	152		52.1	945		56.3
mean (SD)	25.6		4.2	26.2		4.7	25.3			3.8	26		4	26.4		4.7
Education																
Low	265		55.4	110		59.1		146		50		–		997		59.4
Middle	115		24.1	42		22.6		73		25		–		420		25
High	79		16.5	26		14		53		18.2		–		263		15.7
Smoking Status																
Never smoked	321		69.3	147		80.8			–		193		66.1	936		55.7
Quit smoking	92		19.9	23		12.6			–		62		21.2	592		35.2
Current smoker	50		10.8	12		6.6			–		30		10.2	124		7.4
Tumor Characteristics																
In situ/T0	39		8.2	10		5.4		29		9.9		–			NA	
T1	313		65.5	116		62.4		197		67.5		–			NA	
T2	112		23.4	52		28		60		20.5		–			NA	
T3/T4	1		0.2	0		0		1		0.3		–			NA	
Cancer Treatments																
Radiotherapy	478		100	186		100		292		100	292		100		NA	
Tamoxifen	375		78.5		–				–		219		75		NA	
Aromatase inhibitors	95		19.9		–				–		52		17.8		NA	
Living alone	125		26.2	67		36		58		20.1	90		30.8	360		21.4
Comorbidities (Chronic Diseases)																
Depression		NA			NA				NA		43		14.7		NA	
Hypertension	153		32	67		36		86		29.5	148		50.7	635		37.8
Diabetes	31		6.5	23		12.4		8		2.7	25		8.6	100		6
Stroke		NA			NA				NA		9		3.1	31		1.8

^*^Only controls recruited from the Rhine-Neckar-Karisruhe region [33, 34] were include for better comparability

### Longitudinal analysis on the change of HRQoL from RT to 10-year follow-up

**Fig. 1:**
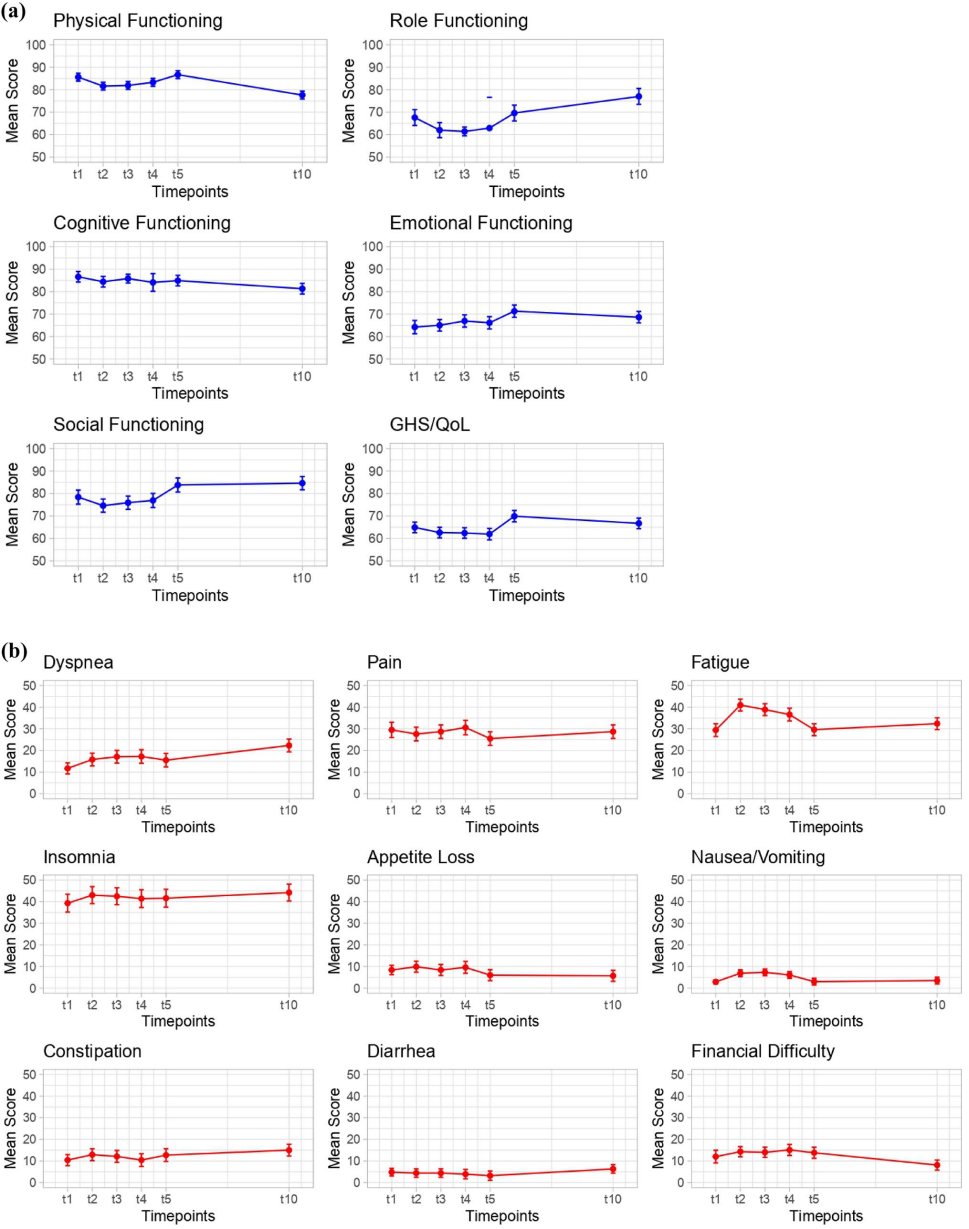
Mean EORTC QLQ-C30 scores of **a** Global Health Status/Quality of Life (GHS/QoL), functional and **b** symptom domains of the patient cohort (*N* = 292) at baseline (t1), during radiotherapy (t2, t3), end of radiotherapy (t4), 6 weeks after radiotherapy (t5) and the 10-year follow-up (t10). Higher scores indicated better GHS/QoL and functional performances (1a), but a higher level of symptom severity (1b). The normalized EORTC QLQ-C30 scale ranges from 0 to 100

Figure 1 shows the changes of GHS/QoL and functional performances (Fig. 1a) as well as the symptom domains (Fig. 1b) from baseline (pre-RT, t1) through 6 weeks after RT (t5) until the 10-year follow-up (t10) among BC survivors. When evaluating changes between selected time points (Table 2), age-adjusted scores at baseline (t1), 6 weeks after RT (t5) and the 10-year follow-up (t10) were compared. For most functional domains, there was an overall decrease in functional scores during the acute treatment period (t2 to t4) after baseline (t1), with a recovery at 6 weeks after RT (t5), which was followed by deteriorations (e.g., physical functioning) or stable scores at 10 years post-diagnosis (t10) for most functional domains except for role functioning. A significant improvement was observed between t1 and t5 for GHS/QoL, emotional and social functioning. Role functioning scores significantly improved by 7.4 points during the 10-year follow-up compared to post-RT scores (77.0 vs. 69.6). On the other hand, for GHS/QoL, physical and cognitive functioning, mean scores were significantly lower at t10 compared to t5, with mean score differences of 3.2, 9.1, and 3.6 points, respectively. When comparing baseline (t1) and the 10-year follow-up (t10), a significant improvement was observed for role, emotional and social functioning, whereas physical and cognitive functioning mean scores were significantly lower at t10 compared to t1.

**Table 2:** Mean age-adjusted HRQoL scores and differences over time at baseline (t1), 6 weeks after radiotherapy (t5) and 10 years after diagnosis (t10) in 292 breast cancer patients and age effect on mean score differences between t10 and t5 for selected domains; α = 0.05

	Means of EORTC QLQ-C30 score			Pair-wise p values^a^			Age effect on mean differences between t10 / t5		
	t1	t5	t10	t1 vs. t5	t1 vs. t10	t5 vs. t10	*β* age^b^	*p* value age^c^	*p* value Intercept^d^
Global HRQoL									
GHS/QoL	64.9	69.9	66.7	0.0001***	0.14	0.013*	−0.48﻿	0.02	0.03
Functioning domains									
Physical functioning	85.6	86.7	77.6	0.23	<0.0001***	<0.0001***	−0.47	0.003	0.05
Role functioning	67.6	69.6	77.0	0.28	<0.0001***	0.0001**	−0.65	0.02	0.001
Cognitive functioning	86.6	84.9	81.3	0.18	<0.0001***	0.0037**	−0.26	0.19	0.02
Emotional functioning	64.2	71.3	68.6	<0.0001***	0.001**	0.053	–	–	–
Social functioning	78.4	83.8	84.6	0.0008***	<0.0001***	0.6	–	–	–
Symptom domains									
Dyspnea	11.7	15.5	22.3	0.017*	<0.0001***	<0.0001***	0.34	0.21	0.0005
Pain	29.5	25.5	28.7	0.01*	0.62	0.05	–	–	–
Fatigue	29.4	29.6	32.4	0.9	0.026*	0.05	–	–	–
Insomnia	39.3	41.6	44.2	0.26	0.01*		–	–	–
Appetite loss	8.4	6.0	5.7	0.07	0.03*	0.83	–	–	–
Nausea vomiting	2.9	3.0	3.5	0.95	0.5	0.94	–	–	–
Constipation	10.4	12.7	15.0	0.12	0.001**	0.12	–	–	–
Diarrhea	4.8	3.2	6.3	0.13	0.12	0.003**	0.4	0.008	0.01
Financial difficulty	12.0	13.8	8.1	0.14	0.001**	<0.0001***	0.51	0.02	0.0006

*GHS/QoL Global* Health Status/Quality of Life

^a^Mean age-adjusted score differences between selected time-points from linear mixed models

^b^Results from linear regression models on the effect of baseline age on mean differences between t10 and t5

^c^*p*-values for the age effect

^d^*p*-values for the intercept with mean age constant (significance of mean differences)

^*^p < 0.05, **p < 0.01, ***p < 0.001

Regarding the symptom domains, dyspnea scores increased by 10.6 points from 11.7 at t1 to 22.3 at t10. Pain scores were relatively stable between 29.5 at t1 and 28.7 at t10, respectively. Fatigue had a peak during RT (41.0 at t2) but returned to baseline level (32.4 at t10). Insomnia scores increased and remained relatively stable after baseline with mean scores of 44.2 at t10. Appetite loss, nausea/vomiting, constipation and diarrhea scores remained relatively stable at low levels of up to 15.0. Financial burden significantly improved from t5 to t10, at low levels though.

The specific effect of age at baseline and its relationship with previously observed significant differences between t10 and t5 was also evaluated. For GHS/QoL, physical and role functioning, baseline age was associated with worsening in functional scores between t5 and t10, with statistically significant differences in mean scores between time points after the age adjustment. For diarrhea and financial difficulty, baseline age was associated with an increase in mean scores between t5 and t10. Similar to the functional domains, mean differences were also still significant between time points after adjustment for baseline age. For cognitive functioning and dyspnea, age was not significantly associated with mean differences between t5 and t10.

Patients with recurrences/metastasis had generally lower GHS /QoL, lower functional and higher symptoms scores than patients without disease progression (Fig. S2).

**Fig. 2:**
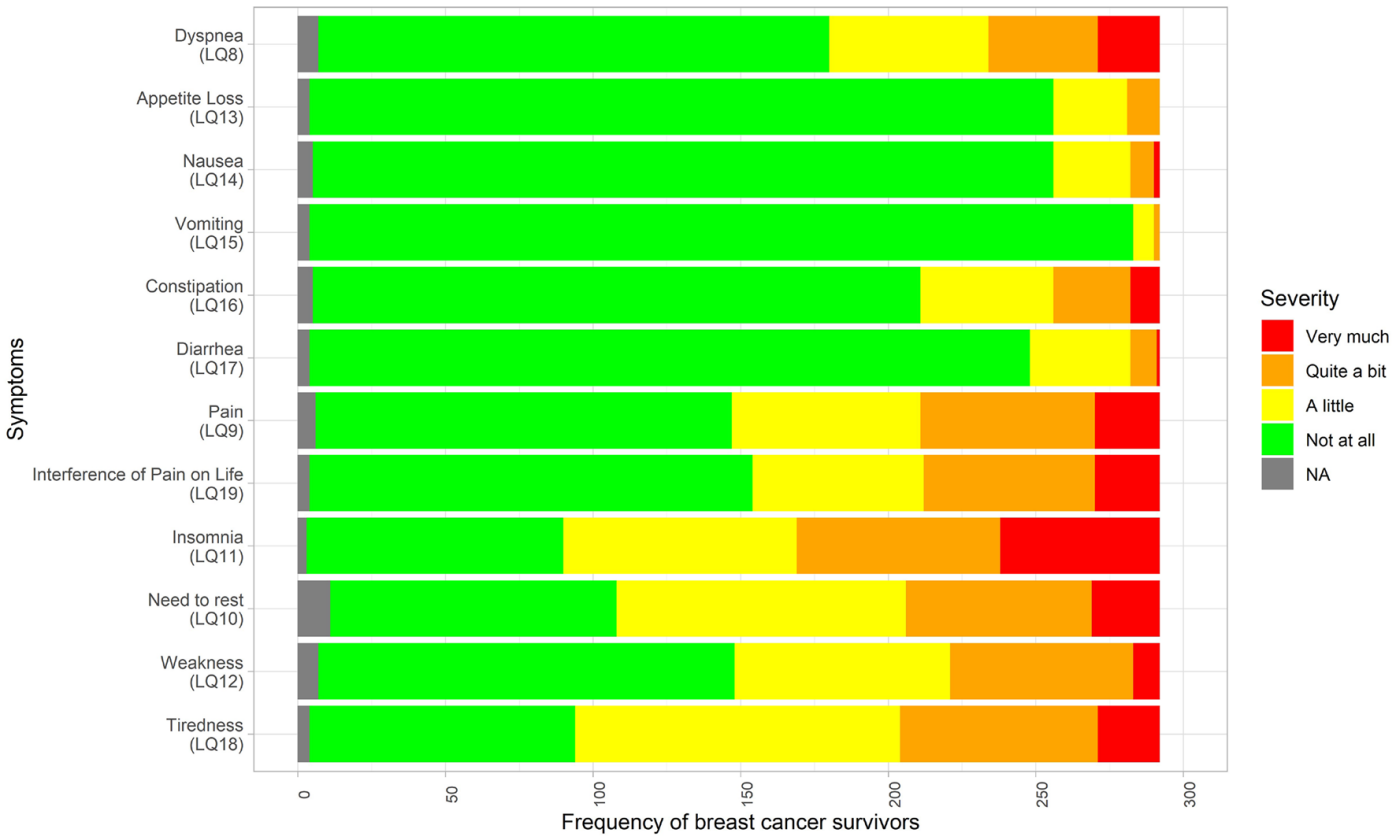
Frequency of patients reporting EORTC QLQ-C30 symptoms at the 10-year follow-up. Original response options are presented varying from “not at all” to “very much”. The fatigue (LQ10 need to rest, LQ12 weakness, LQ18 tiredness) and pain (LQ9 pain, LQ19 pain interfering with daily life) dimensions consist of more than one question

Sleep problems, fatigue, pain and dyspnea were the most prevalent symptoms in BC survivors at the 10-year follow-up (Fig. 2). About a third to almost half of patients reported moderate to severe fatigue and sleep problems, respectively.

### Comparison of HRQoL with the control population at the 10-year follow-up

**Fig. 3:**
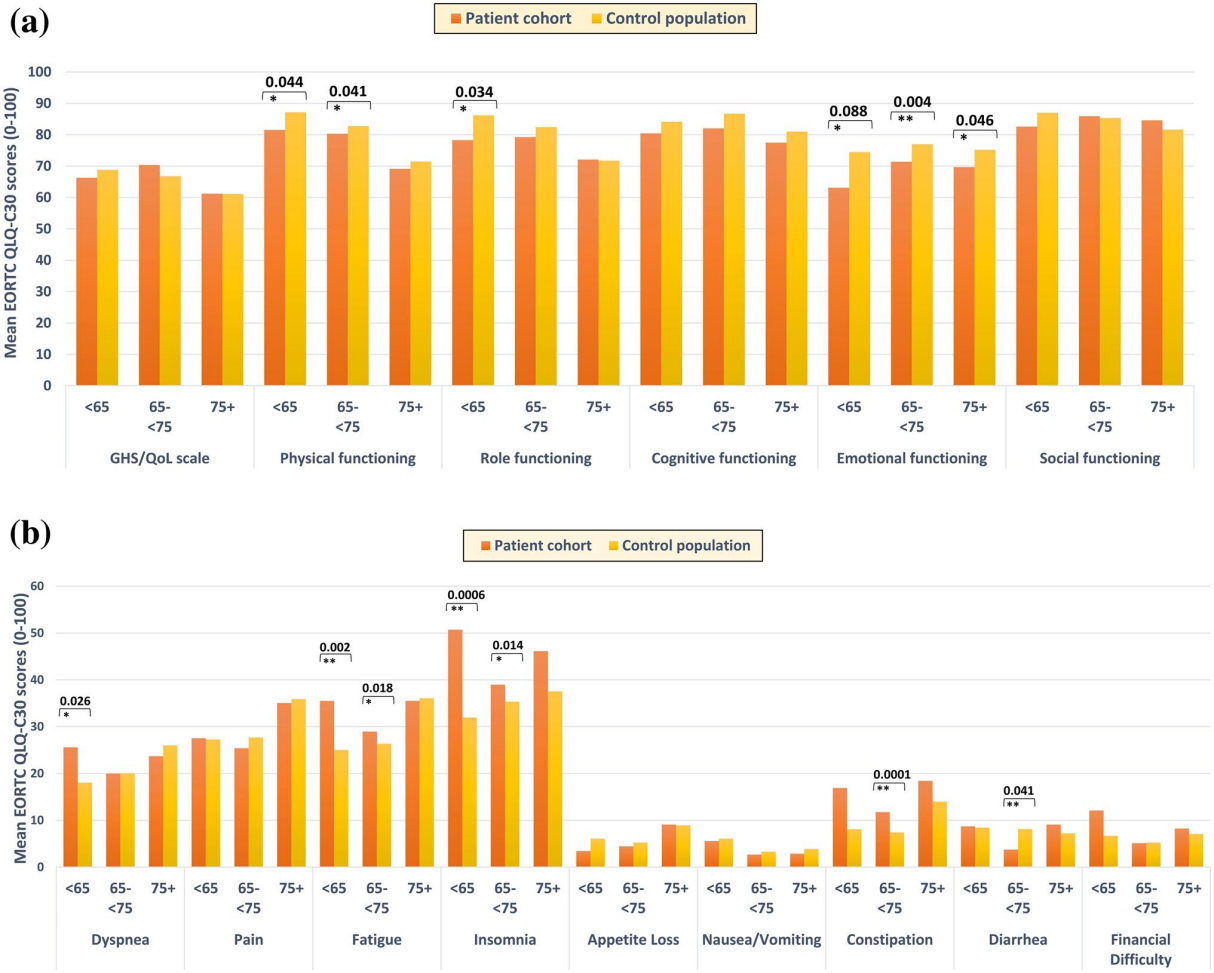
Differences of EORTC QLQ-C30 scores in **a** Global Health Status/Quality of Life (GHS/QoL), functional and **b** symptom domains between breast cancer patients and cancer-free controls at the 10-year follow-up by age stratification; p values were obtained from non-parametric Wilcoxon rank-sum test and significant differences were indicated by asterisks (*p <0.05, **p<0.01,***p< 0.001)

The characteristics of the patient cohort as well as the control population at the 10-year follow-up were summarized in Table 1. No significant differences in GHS/QoL in BC survivors and the control population were observed at the 10-year follow-up for the different age groups. However, deficits in physical and emotional functioning and in certain symptoms such as dyspnea, fatigue, sleep problems and constipation were more prevalent in BC survivors than controls (Fig. 3; Table S1). Detriments in functional performance and symptom burden occurred predominantly in younger survivors. Compared to controls of comparable age, survivors under 65 years of age reported significantly worse physical (81.6 vs. 87.1), role (78.3 vs 86.2) and emotional functioning (63.1 vs. 74.5), as well as higher burden of dyspnea (25.6 vs. 18.0), fatigue (35.5 vs. 25.0) and sleep problems (50.7 vs. 32.0). The prevalence of psychosocial dysfunction and symptoms among younger survivors showed that younger patients were at greater risk of lower HRQoL years after RT. Patients aged ≥ 75 years reported similar functional performance and symptom burden compared to the control population except for a deficit in emotional functioning (difference of 5.5 points). Middle aged patients reported worse physical (2.5 points) and emotional functioning (5.6 points), and more severe fatigue and sleep problems compared to controls (difference of 2.6 and 3.6 points, respectively). Besides, gastrointestinal problems mainly affected the middle-age group.

### Determinants of HRQoL in long-term BC survivors (cross-sectional analysis)

Results from multivariable linear regression (Table S2) showed that a poorer GHS/QoL and physical functioning was associated with being overweight. Living with others was associated with better GHS/QoL and physical functioning, lower levels of dyspnea and pain at the long-term follow-up, while patients above 65 years had decreased levels of dyspnea. Depression was found to be associated with lower GHS/QoL, worse functioning, and a higher level of pain, fatigue, insomnia, gastrointestinal problems, and financial difficulty. Besides, hypertension was associated with more severe dyspnea and fatigue as well as worse physical and role functioning. Other side effects such as pain/lymphedema and fibrosis were associated with worse physical and social functioning, respectively.

## Discussion

In this distinct cohort of breast cancer patients treated with radiotherapy after breast-conserving surgery, we found that GHS/QoL declined during RT, but returned to baseline levels after completion of RT. In long-term survivors more than 10 years post-RT, GHS/QoL was not found to be significantly different to that of controls of comparable age. However, HRQoL impairment was found for certain specific HRQoL domains, in particular survivors younger than 65 years reported poorer psychosocial functioning and substantially more fatigue and sleep problems than controls of comparable age.

### Longitudinal analysis of HRQoL in BC survivors

Few studies have examined the change in HRQoL scores in BC survivors [4, 10–14, 16, 17, 19] as in this study with repeated measurements not only during and 6 weeks after post-operative RT but also with measurements up to 10 years after treatment. Previous studies analyzing HRQoL of BC patients during and after RT [37, 38] did not find significantly worsened GHS/QoL in BC patients although fatigue and most symptoms increased during RT, which might be related to side effects of treatment. On the whole, GHS/QoL scores at 5–10 years of follow-up have been reported to be comparable to baseline level [4, 12, 16, 17, 19, 39]. Therefore, our findings based on longitudinal data even after > 10-year follow-up are in line with previously reported results.

At 10-year follow-up, the BC survivors in our study had better role, emotional and social functional performances compared to baseline, but a worse physical and cognitive functioning. When compared to 6 weeks after RT, they showed only improvement in role functioning and restrictions in physical and cognitive functioning. Although increasing age was found to be significantly correlated with worsening of both functional and symptom domains between t5 and t10, mean differences were still significant after age adjustment, suggesting that, although aging plays an important role in long-term HRQoL differences, it is not sufficient to explain improvement or worsening of HRQoL scores over time. Previous studies also found that BC survivors often gradually recovered from most functional restrictions in the long term [4, 12, 19, 40]. Nonetheless, on the symptom domains, our results confirmed that fatigue and sleep problems may persist for years after treatment [41–44]. The level of pain after RT did not show significant differences compared to 10 years and remained stable during the treatment period, whereas a significant improvement in pain over time was reported by another study [45]. Dyspnea as a potential effect of RT was reported more often by younger BC survivors at the 10-year follow-up in our study and was also the HRQoL symptom domain with the largest deterioration from baseline to the 10-year follow-up. Several comorbidities such as hypertension rather than aging itself were associated with higher levels of dyspnea in our determinants analysis. A previous study on long-term HRQoL (10 years) also found that young BC survivors experience the greatest deterioration in multiple HRQoL domains during follow-up compared to other age groups, and among symptom domains, the greatest differences with a reference population were found for dyspnea and fatigue [17]. Similar findings have also been previously reported for dyspnea when comparing BC survivors and the German reference population [33], although other studies have reported similar late dyspnea levels between BC survivors and a reference group [44, 46]. Most of the significant differences observed when comparing functional and symptoms mean scores over time were associated with “little change” according to the minimal clinically important difference definition proposed by Osoba et al. [47] (i.e., differences of about 5 to 10 points).

### Cross-sectional comparison of HRQoL with controls at the 10-year follow-up

To account for the impact of aging, we compared the HRQoL of the long-term BC survivors more than 10 years after diagnosis to a control population that was of comparable age but without a breast cancer diagnosis at baseline and were also followed up for about 10 years. The GHS/QoL in the long-term BC survivors was comparable to that of the controls, which is in line with several previous studies [4, 12, 13, 19]. Yet both our findings and that of others found significant restrictions in certain domains, most commonly for fatigue [18, 20, 44], sleep problems [16, 20], and emotional functioning [18, 19, 44], providing clear evidence that HRQoL may be persistently impaired in cancer patients in the long run. In particular, long-term survivors younger than 65 years at 10-year follow-up reported poorer psychosocial related HRQoL performances in our study, including significantly higher levels of fatigue and sleep problems than controls of comparable age. For emotional functioning, fatigue and sleep problems in survivors under 65 compared to controls, the observed differences can be characterized as “moderate clinically meaningful differences” (i.e., 10–20 points) according to Osaba et al. [47] and Cocks et al. [48].

Physical functioning has been reported by patients below 80 years to be significantly poorer than their age-matched reference groups, but they were comparable to that of healthy controls in the oldest age group above 80 years [16]. We observed a similar pattern in physical functioning, which could be explained by natural deterioration with aging. Deficits in role functioning were apparent in the youngest age group, suggesting that younger BC survivors might have more problems to cope with this difficult life situation and may continue to suffer more even 10 years after diagnosis. Yet detriments in emotional functioning persisted at the 10-year follow-up among our BC survivors compared to controls for all age groups. In BC survivors of the MARIE study, however, the youngest age group suffered greater detriments in emotional functioning compared to controls [19]. Our results suggest that emotional well-being might be a problem for BC survivors across a wide age range. In contrast to previous findings showing differences in financial difficulties [12, 21, 44], pain [20], and cognitive functioning [18, 19, 44], there was no significant difference between patients and controls in these domains in any of the age groups in our study. Yet dyspnea was found to be significantly more severe among the youngest patient age group compared to controls.

We confirmed a number of determinants for an impaired long-term quality of life of breast cancer survivors such as younger age [16], obesity [25], or certain comorbidities like depression [26]. In addition to RT and cancer surgery, other parameters like endocrine therapy or BC treatment-related symptoms such as lymphedema or pain were significantly associated with higher burden of certain quality of life domains. In addition, other factors like anxiety, limited mobility of shoulder joint, or factors unrelated to the BC diagnosis may also play a role for impaired long-term HRQoL.

The differences in findings among studies might be explained by different age structures of the study populations and different inclusion criteria. Our patient cohort tended to have a younger age structure and a wider age range compared to other studies, and younger survivors have considered cancer as a bigger threat and have been more affected in social aspects [23]. Furthermore, all BC patients included in this study only received adjuvant RT, while other studies included patients who had also received chemotherapy; thus, the therapy-related symptom burden could be different.

### Strengths and limitations

We assessed HRQoL in a unique cohort of breast cancer patients who received only radiotherapy after breast-conserving surgery but not chemotherapy. The study was focused on side effects after radiotherapy and attempted to minimize “contamination” due to chemotherapy. Although RT techniques have improved in the last two decades (e.g., intensity-modulated/image-guided RT), resulting in fewer treatment-related adverse effects due to less damage to surrounding tissues, long-term QoL data in the post-treatment period are scarce. One study from the Netherlands also limited their study population to breast cancer patients treated with RT and without chemotherapy, yet the follow-up period was only 3 years [45]. Second, our study is one of the few studies with prospective multidimensional HRQoL assessments that comprise a baseline (pre-RT) and multiple follow-up evaluations during and post-RT treatment over a period of more than 10 years. Third, we compared the long-term HRQoL of the breast cancer survivors to a group of unaffected women with comparable length of follow-up to account for an aging effect. As comparison group, we used a large control population from the same geographic region [33, 34] rather than German general population norm data [49] that has been used in other studies [4, 11, 17, 20, 33, 43, 45, 50] in order to account for regional differences and increase statistical power. Finally, unlike other studies, e.g., [19, 44], there was no age restriction in our study to capture a wider spectrum of patients and further enhance the representativeness.

The analysis has some limitations: Because of the eligibility criteria of the parent study design, the results might not be generalizable to patients who have received chemotherapy as part of their cancer treatment, acknowledging that chemotherapy is an important risk factor for impaired HRQoL. However, findings were similar between our study and studies including chemotherapy patients. The response pattern of our study might have caused an underestimation of the true difference between survivors and controls as those who had passed away or did not participate due to other reasons tended to have worse health conditions (severe symptoms or worse functional performances) which might lead to a lower HRQoL (survivorship bias). With this loss of patients, the sample size was greatly reduced, entailing a loss of statistical power when analyzing subgroups. Overestimation of the observed HRQoL could also have occurred since patients with poor health might be less likely to participate in the study at baseline and also at the follow-ups. Due to the nature of the collected data (both the BC patient cohort and the control population), the data on the baseline HRQoL measures for controls and the HRQoL data for BC patients prior to the conserving surgery or diagnosis are not available. There were 68 controls who had a previous (non-breast) tumor at recruitment and 31 who developed cancer during follow-up. We did not exclude these individuals from the analysis to better represent the general population. With this approach, we could have underestimated the differences between patients and controls. Due to the age restriction at recruitment in the MARIE study (50–74 years), the age distribution of patient cohort and control population was not balanced, and the controls were primarily postmenopausal, while there was no age restriction at recruitment in ISE study, which could reduce the generalizability in the lowest age group. Besides, for the cross-sectional determinants analysis, additional factors, in particular other comorbidities than depression, hypertension, diabetes, and stroke which we accounted for, may have a relevant impact on HRQoL.

## Conclusion

In summary, GHS/QoL declined during RT, but returned to baseline levels after completion of RT and was not significantly different to a control population after 10 years. However, deficits were reported in specific functional and symptom domains such as fatigue, sleep problems, or emotional burden, in particular of younger survivors. The study results demonstrate that symptom burden as well as detriments in functional domains in long-term BC survivors may persist for more than 10 years after cancer diagnosis, emphasizing the importance of long-term follow-up care beyond the usual 5-year routine post-treatment care with primary focus on tumor recurrence. As cancer survivors often suffer from multiple symptoms/chronic diseases [51] and fatigue was shown to be associated with worse survival in cancer patients [2], survivorship care should aim to identify BC survivors at risk of sleep problems, fatigue, psychosocial, and other health needs, taking into account age, treatment-related aspects, and personal risk factors. Targeted psychosocial interventions and supportive actions (e.g., counseling) should be offered in early phases where needed, particularly to younger patients, to improve HRQoL and symptom burden and prevent manifestation in the future.

## Supplementary Information

Below is the link to the electronic supplementary material.

Supplementary file1 (PDF 1406 KB)

## Data Availability

The datasets generated and/or analyzed during the current study are not publicly available but are available from the corresponding author on reasonable request (acceptance of a project proposal and signature of a data transfer agreement).
