# Supplementary material for: Longitudinal changes of health-related quality of life over 10 years in breast cancer patients treated with radiotherapy following breast-conserving surgery
Source: Qual Life Res. 2023 Apr 24;32(9):2639–52. doi: 10.1007/s11136-023-03408-y (PMC10393846; doi:10.1007/s11136-023-03408-y)
Supplement: Supplementary file 1 — Supplementary file1 (PDF 1406 KB) [file 11136_2023_3408_MOESM1_ESM.pdf]

Article: Longitudinal course of health-related quality of life over 10 years in breast cancer patients treated with radiotherapy following breast-conserving surgery

Journal: Quality of Life Research

Authors: Yifeng Gao, Juan Camilo Rosas Romero, Hanna Fink, Sabine Behrens, Jenny Chang-Claude, Petra Seibold

***Corresponding author:**

Dr. Petra Seibold

Division of Cancer Epidemiology

German Cancer Research Center (DKFZ), Im Neuenheimer Feld 581, 69221 Heidelberg, Germany

E-mail: p.seibold@dkfz-heidelberg.de

Section 1. Study cohort characteristics

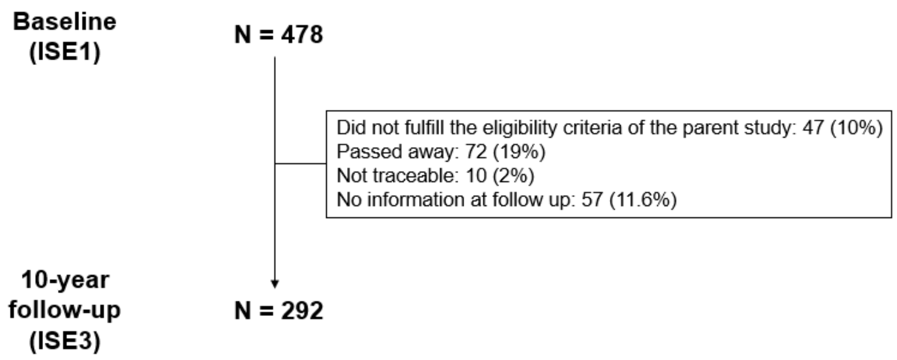

Figure S1. CONSORT diagram of breast cancer patient cohort.

Section 2. Subgroup analysis

(a)

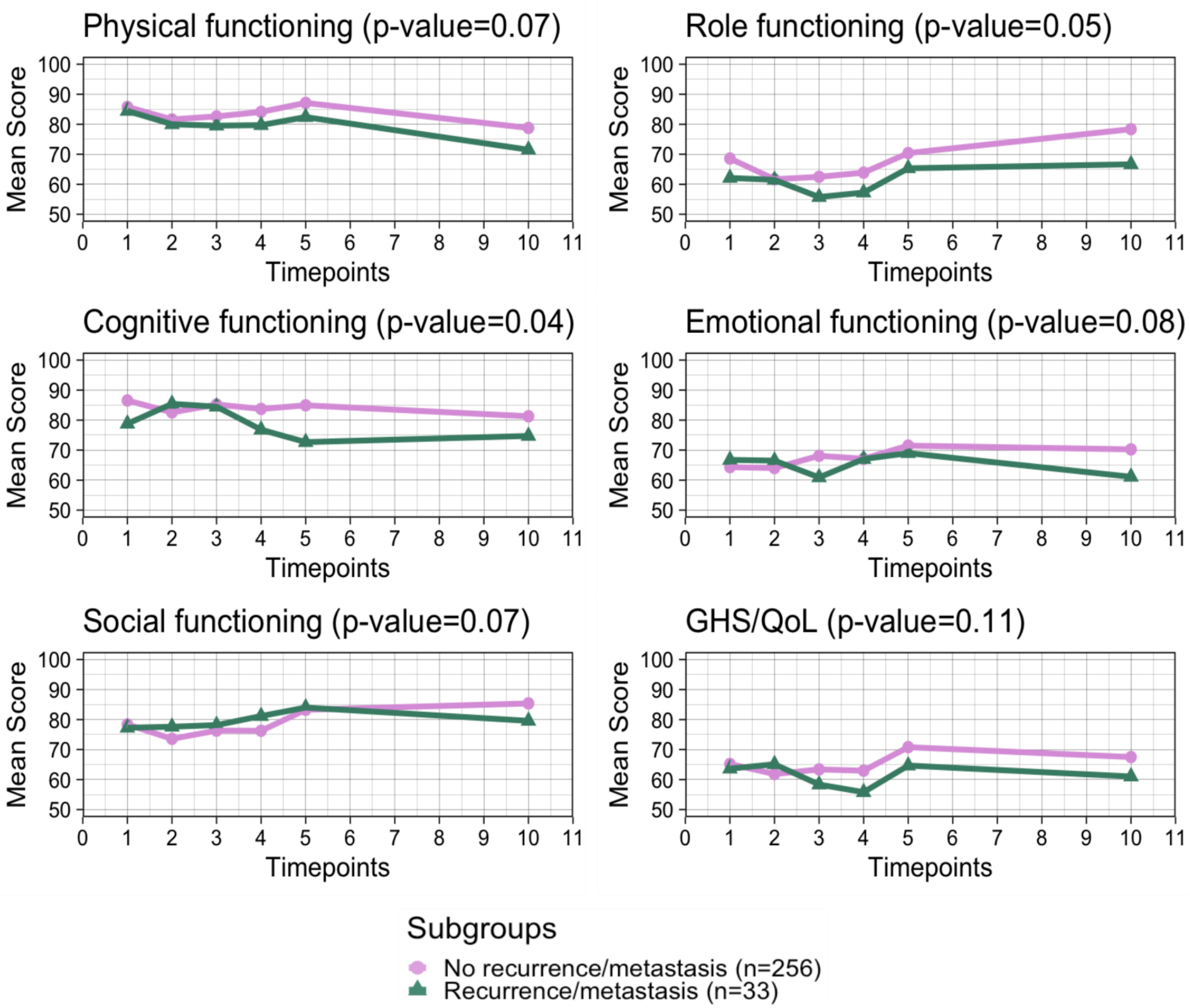

Figure S2. *Cont.*

(b)

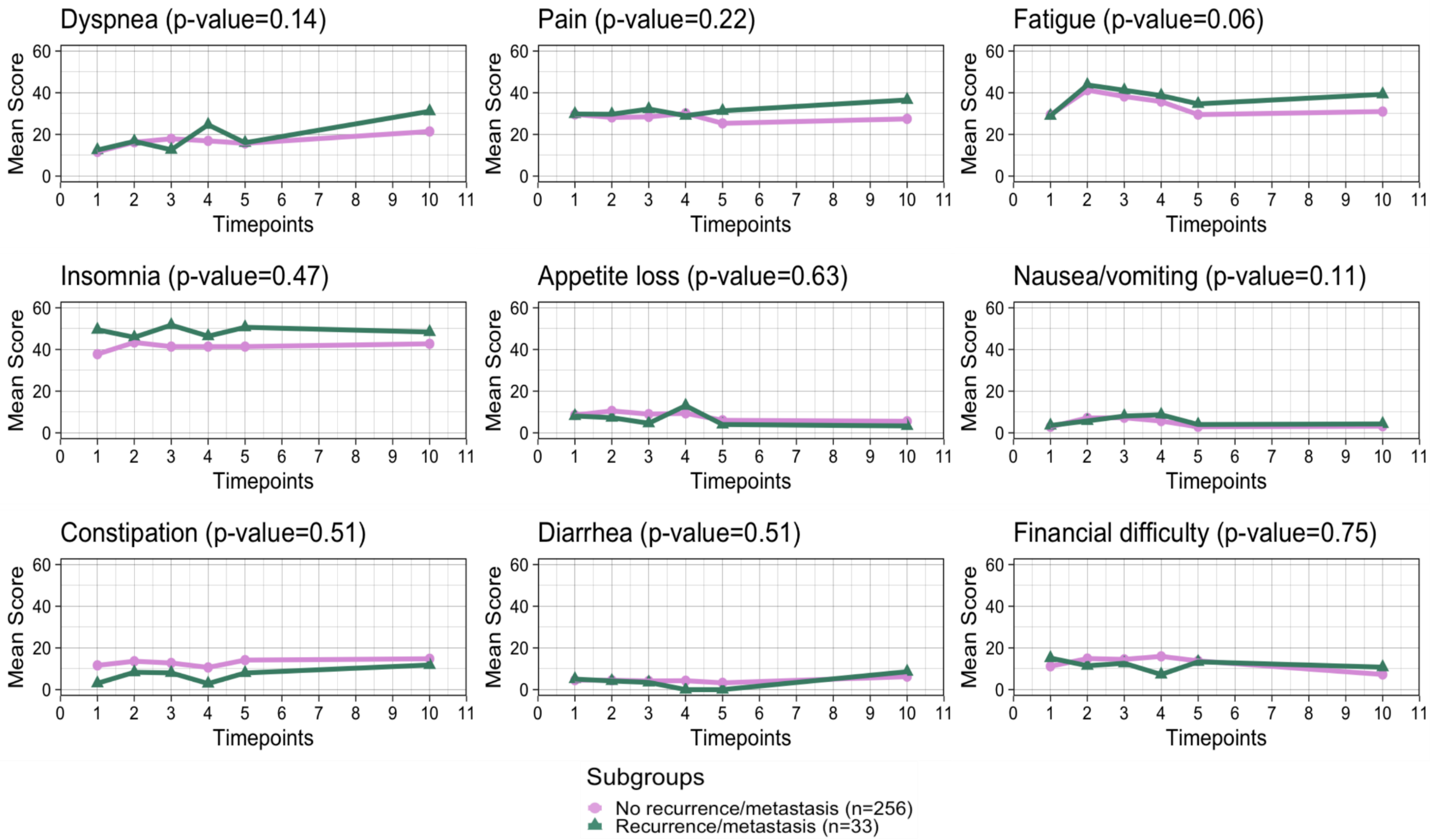

Figure S2. Mean QLQ-C30 scores for (a) Global Health Status/Quality of Life (GHS/QoL), functional and (b) symptom domains from baseline to 10 years after diagnosis by recurrence/metastases status. P-values indicate the heterogeneity at the 10-year time-point. No statistical significance at the previous time-points.

Section 3. Cross-sectional comparison between breast cancer patients and control population

Table S1. Wilcoxon rank-sum test for differences of EORTC QLQ-C30 scores in Global Health Status/Quality of Life (GHS/QoL), functional and symptom scales/items between breast cancer patients and control population at the 10-year follow-up after age-stratification

	Wilcoxon Test for Differences between ISE3 & MARIE Control								
	**Age < 65 (N=169)**			**65 <= Age < 75 (N=956)**			**Age >= 75 (N=841)**		
	means of EORTC QLQ-C30 score		p-value	means of EORTC QLQ-C30 score		p-value	means of EORTC QLQ-C30 score		p-value
	patients (N=69)	controls (N=100)		patients (N=145)	controls (N=811)		patients (N=78)	controls (N=763)	
Global HRQoL									
GHS/QoL	66.30	68.79	0.61	70.36	66.81	0.09	61.22	61.13	0.94
Functional domains									
Physical functioning	81.55	87.13	**0.044***	80.34	82.79	**0.041***	69.12	71.43	0.34
Role functioning	78.26	86.20	**0.034***	79.25	82.46	0.08	72.08	71.75	0.97
Cognitive functioning	80.43	84.17	0.51	82.05	86.70	0.14	77.49	81.04	0.65
Emotional functioning	63.08	74.50	**0.008****	71.42	76.97	**0.004****	69.70	75.23	**0.046***
Social functioning	82.61	87.00	0.14	85.92	85.33	0.74	84.62	81.67	0.61
Symptom domains									
Dyspnea	25.60	18.03	**0.026***	20.00	20.02	0.87	23.68	26.02	0.57
Pain	27.54	27.27	0.85	25.41	27.69	0.63	35.04	35.90	0.69
Fatigue	35.51	25.03	**0.002****	28.94	26.37	**0.018***	35.50	36.07	0.72
Insomnia	50.72	31.97	**0.0006*****	38.97	35.33	**0.014***	46.15	37.54	0.06
Appetite loss	3.43	6.06	0.65	4.43	5.24	0.55	9.09	8.92	0.70
Nausea/vomiting	5.56	6.06	0.61	2.68	3.28	0.93	2.85	3.88	0.92
Constipation	16.91	8.08	0.07	11.74	7.40	**0.0001*****	18.42	13.99	0.27
Diarrhea	8.70	8.42	0.41	3.76	8.15	**0.041***	9.09	7.21	0.36
Financial difficulty	12.08	6.67	0.12	5.13	5.23	0.70	8.23	7.07	0.71

α=0.05; *p<0.05, **p<0.01, ***p<0.001

Abbreviations: GHS/QoL: Global Health Status/Quality of Life

Section 4. Multiple linear regression model

Table S2. Multiple linear regression models on Global Health Status/Quality of Life (GHS/QoL), functional and symptom domains in breast cancer patients at the 10-year follow-up

(a) Functional domains and GHS/QoL (model with best fits)

		Functional domains & GHS/QoL (0 - 100)											
		Physical Functioning (R^2 = 14.8)		Role Functioning (R^2 = 6.6)		Cognitive Functioning (R^2 = 5.4)		Emotional Functioning (R^2 = 9.9)		Social Functioning (R^2 = 8.5)		GHS/QoL (R^2 = 7.9)	
**Covariates**	N	ß	(95% CI)	ß	(95% CI)	ß	(95% CI)	ß	(95% CI)	ß	(95% CI)	ß	(95% CI)
**Age**													
< 65	69												
65 - <75	145												
> 75	78												
**Living with others**	202	**8.35**	**(2.44, 14.25)***										
**BMI >= 25 kg/m2**	152	**-6.33**	**(-11.94, -0.72)***									**-6.46**	**(-12.2, -0.74)***
**Smoking Status**													
Never smoked	193			Ref.		Ref.							
Quit smoking	62			0.74	(-8.37, 9.85)	-0.66	(-7.95, 6.62)						
Current smoker	30			9.44	(-1.83, 20.71)	**10.17**	**(0.71, 19.62)***						
**Other adjuvant treatments**													
Aromatase inhibitors	52	-6.1	(-12.99, 0.81)										
Tamoxifen	219												
**Chronic Diseases**													
Depression	43	-6.88	(-14.3, 0.54)	**-13.67**	**(-23.28, -4.1)****	**-8.92**	**(-16.84, -0.99)***	**-20.28**	**(-28.82, -11.73)*****	-7.95	(-16.82, 0.92)	**-11.46**	**(-18.92, -4.0)****
Hypertension	148	**-8.12**	**(-13.77, -2.5)****	**-10.54**	**(-17.73, -3.34)****			-5.28	(-11.69, 1.14)	-3.95	(-10.47, 2.58)	**-6.33**	**(-12.06, -0.59)***
Stroke	11					**-20.97**	**(-35.43, -6.50)****	**-17.83**	**(-35.65, -0.02)***				
Diabetes	25												
**Recurrence, metastases and other tumors**	33			-7.43	(-18.33, 3.46)			-3.85	(-13.92, 6.21)	-9.97	(-22.24, 2.3)	-5.57	(-14.1, 2.92)
**Other Symptoms**													
Pain/Lymphedema	19	**-10.29**	**(-20.3, -0.27)***							**-18.1**	**(-30.87, -5.28)****		
Fibrosis	87									**-8.1**	**(-15.1, -1.13)***		

This table included all covariates after AIC selection; covariates were assesed at the 10-year follow-up; N refers to the overall frequency for every level; *p<0.05, **p<0.01, ***p<0.001

Abbreviations: GHS/QoL: Global Health Status/Quality of Life; BMI: Body mass index

(b) Symptom domains (model with best fits)

		Symptom domains (0-100)																	
		Dyspnea (R^2 = 20.2)		Pain (R^2 = 5.4)		Fatigue (R^2 = 6.5)		Insomnia (R^2 = 2.9)		Appetite Loss (R^2 = 4.9)		Nausea/Vomiting (R^2 = 4.2)		Constipation (R^2 = 2.1)		Diarrhea (R^2 = -0.1)		Financial Difficulty (R^2 = 11.1)	
**Covariates**	N	ß	(95% CI)	ß	(95% CI)	ß	(95% CI)	ß	(95% CI)	ß	(95% CI)	ß	(95% CI)	ß	(95% CI)	ß	(95% CI)	ß	(95% CI)
**Age**																			
< 65	69	Ref.								Ref.									
65 - <75	145	**-12.2**	**(-22.62, -1.79)***							1.29	(-3.29, 5.87)								
> 75	78	**-19.5**	**(-32.6, -6.4)****							**6.85**	**(1.44, 12.25)***								
**Living with others**	202	**-17.1**	**(-26.72, -7.49)*****	**-10.7**	**(-19.73, -1.57)***														
**BMI >= 25 kg/m2**	152																		
**Smoking Status**																			
Never smoked	193																		
Quit smoking	62																		
Current smoker	30																		
**Other adjuvant treatments**																			
Aromatase inhibitors	52	**16.43**	**(5.4, 27.47)****							1.68	(-2.91, 6.27)								
Tamoxifen	219			**11.32**	**(0.16, 22.5)***														
**Chronic Diseases**																			
Depression	43	7.26	(-4.12, 18.64)	**11.3**	**(0.54, 22.05)***	**14.83**	**(6.13, 23.53)*****	**16.01**	**(3.71, 28.30)***	**7.83**	**(2.76, 12.89)*****	**6.3**	**(2.68, 9.9)*****	**11.44**	**(2.48, 20.41)***			**16.35**	**(10.39, 22.32)*****
Hypertension	148	**15.11**	**(6.26, 23.95)*****	6.37	(-1.99, 14.74)	**8.44**	**(1.88, 15.0)***	8.35	(-0.81, 17.51)			1.06	(-1.64, 3.76)					3.27	(-1.18, 7.73)
Stroke	11	**25.18**	**(1.8, 48.57)***									4.1	(-2.98, 11.13)						
Diabetes	25	**18.72**	**(4.8, 32.64)****													2.88	(-3.79, 9.54)	5.46	(-2.11, 13.07)
**Recurrence, metastases and other tumors**	33	7.22	(-6.14, 20.59)	6.87	(-5.73, 19.47)	6.95	(-3.1, 17.0)												

This table included all covariates after AIC selection; covariates were assesed at the 10-year follow-up; N refers to the overall frequency for every level; *p<0.05, **p<0.01, ***p<0.001

Abbreviations: BMI: Body mass index
